# The Use of Tobacco

**Published:** 1850-12

**Authors:** 


### THE USE OF TOBACCO.

*The Medical Examiner* contains the following remarks:

"We have been requested by a much respected friend, to lay before our readers, some queries recently addressed to him by an English Surgeon, in relation to the influence of Tobacco upon the health, in the hope that some of our readers would be able to afford the desired information. He says: "many circumstances of late have occurred, in which I have seen the most injurious effects of the use of tobacco upon the nervous, circulatory, and digestive functions. A friend of mine became a perfect hypochondriac by the use of snuff, and was at once relieved by leaving it off. Upon returning to the use of it, he again suffered as before, and was again relieved by ceasing to take it. Here there was no doubt. Another gentleman was covered with an eruption resembling psoriasis, from head to foot, and got well immediately when he left off the use of snuff. Three times he suffered a relapse upon taking snuff, and was cured by leaving it off. In many smokers, I may say all, I have found heart disease or confirmed dyspepsia. If you can help me with any statistic accounts of disease of the heart and arteries, of brain and nervous system, and of the stomach and chylopoietic viscera, and cancer of the mouth and lips, I should feel greatly obliged. If to these you could add any data of the use of tobacco by the sufferers, it would greatly enhance their value.'

"The subject is a deeply interesting one, and we trust that out of the large experience of our readers, something may be gained to illustrate the effects of this agent upon the economy."

We should not suppose it necessary to wander very far from Philadelphia to ascertain the evil effects of the use of tobacco. It has not been many years since Prof. Chapman published a remarkably instructive case on this subject. The victim was a member of Congress, and a practicing lawyer, who, from immoderate indulgence in the use of the weed, using it only in three ways, had grown so timid that he was frightened at the sound of his own voice, in attending to his congressional duties, and excessively alarmed by the slightest noises. He was the victim of a serious derangement of the nervous system. By discontinuing the use of tobacco, he was effectively and speedily relieved.

In the *Boston Medical and Surgical Journal*, in 1845, Dr. Shipman detailed a number of cases, arising from the use of tobacco, which possess unusual interest. We shall not note these details, although they are very instructive.

In 1846, Dr. Laycock laid before the British Association, a communication in which was a detail of the various evil effects of the use of tobacco. Dr. Wright's views of the physiological effects of tobacco, contained in the same communication, cover the entire ground occupied by the subject. These various sources of information are very complete, and volumes could scarcely throw any more light on the inquiry than may be found in the papers to which we refer. Other cases may corroborate the facts, but can scarcely make them more intelligible.

We presume there is no part of this pill-taking and tobacco-consuming country that does not furnish many deplorable examples of the evil effects of the use of tobacco. We have seen many instances of the almost entire loss of memory; many where the mental and physical energies were nearly destroyed; numerous examples of dyspepsia, diarrhea, and continued headache, that were directly traceable to the use of tobacco, and which were relieved and recuperated by its discontinuance.

We shall present only two cases illustrative of these facts.

Some years ago, Mr. C. D. an old and highly respectable gentleman of Louisville, was the subject, for eighteen months, of severe attacks of diarrhea, which almost invariably came on at night, about 2 o'clock, A. M. He tried various means for the relief of this troublesome symptom, and dieted himself with such strictness, as to satisfy both him and the writer, that the disease was not caused by any improper food. At length, we informed him that his use of tobacco was the cause of his troublesome attacks. He at once abandoned the tobacco, and, after that, though we had been called to him about once a week for eighteen months, and almost always in the night, while he used the tobacco, nearly two years passed without a single return of the diarrhea. At the end of that time, we were summoned to him again at his old hour of the night. He was at once charged with being in the condition of the Apostle Peter's sow, that "was washed and returned to wallowing in the mire," and he confessed that he had recently resumed the use of tobacco. Upon the abandonment of the indulgence, the diarrhea left him, and has not troubled him since.

Six years ago, the writer of these remarks, while chewing tobacco immoderately, undertook to add the the smoking of cigars to his other accomplishment, in this line. A violent attack of diarrhea, far more painful than anything of the kind he had ever experienced before, was the result. This was not ascribed to the proper cause, until "line upon line, and precept upon precept" were enforced by oft repeated attacks. The curling smoke narcotised the faculties of reflection for a considerable length of time, but the diarrhea was at length so certainly traced to the cigars, that the burning incense was abandoned, and with it, the liability to diarrhea disappeared. The curious facts of this case were that the constitution was inured, if constant chewing could inure it, to the use of tobacco, and, in no instance, was there a longer interval than fifteen minutes, between the smoking and the diarrhea. The connection between the smoking and the diarrhea has often been tested, as a matter of curiosity, since the abandonment of the habit of smoking, and the cause and effect are always about fifteen minutes apart. The curiosity has been satiated, and will remain so.

There is no doubt of the fact that the immoderate use of tobacco produces effects frequently that are not far behind the deplorable results of opium eating. It is by no means a very uncommon occurrence to see cases of tobacco indulgence that have no very remote resemblance to delirium tremens. It is a dangerous luxury, and heavy are the exactions for worship at this shrine.

